# A rare case of critical illness polyneuropathy and literature review

**DOI:** 10.12669/pjms.303.4543

**Published:** 2014

**Authors:** Jiachun Feng, Xinmei Jiang, Shaokuan Fang

**Affiliations:** 1 Jiachun Feng, MD, PhD, Department of Neurology, the First Teaching Hospital of Jilin University, Changchun, China.; 2 Xinmei Jiang, MD, PhD, Department of Neurology, the First Teaching Hospital of Jilin University, Changchun, China.; 3 Shaokuan Fang MD, PhD, Department of Neurology, the First Teaching Hospital of Jilin University, Changchun, China.

**Keywords:** Critical illness polyneuropathy, SIRS, Critical illness myopathy

## Abstract

A 40- year-old Male was admitted to the first hospital of Jilin University with the complaint of 4 days of fever and headache and aggravation of weakness in his lower extremities accompanied with dysuria and disturbance of consciousness for one day. He had tachycardia, tachypnea and elevated white blood cell counts. General status of the patient got better day by day, while weakness and pain in his lower extremities had developed and gradually quadriplegia arose. When intensive care unit history, weaning difficulty from mechanical ventilator, clinical manifestations in intensive care unit associated with SIRS, symmetrical paresis pronounced in distal lower extremities, absence of deep tendon reflexes, evidence of distal sensory impairment, presence of electrophysiologic results indicating axonal sensorimotor polyneuropathy and muscle and nerve biopsy results were taken into consideration, he was diagnosed as critical illness polyneuropathy.

## INTRODUCTION

Critical illness polyneuropathy (CIP) and myopathy (CIM) are complications of critical illness that present with muscle weakness and failure to wean from the ventilator. CIP is a distal axonal sensory-motor polyneuropathy affecting limb and respiratory muscles. Limb involvement is symmetrical; it is most prominent in the lower extremities and can be severe. When less severe, muscle weakness is usually more pronounced distally than proximally.CIP is often preceded by septic encephalopathy. In this disorder, the level of consciousness deteriorates. Because encephalopathy is not usually structural, recovery can be rapid, and difficulty in weaning from mechanical ventilation or obvious weakness of limb movements will be the first signs to be noted during this period. CIM is a primary myopathy that is not secondary to muscle denervation, with distinctive electrophysiological and morphological findings.^1^ The clinical features are often much the same as for CIP, with difficulty in weaning from the ventilator, flaccid limbs, and possible reduction in deep tendon reflexes but, if testable, normal sensation. Herein, we describe a case of CIP with nerve and muscle biopsy.

## CASE REPORT

A 40- year-old Male was admitted to the first hospital of Jilin University with the complaint of 4 days of fever and headache and aggravation of weakness in his lower extremities accompanied with dysuria and disturbance of consciousness for one day. He was diagnosed as having Tuberculous (TB) meningitis and received anti-TB treatment for 25 days. He had stayed in intensive care unit due to metabolic acidosis and respiratory failure for almost a month. The patient wasn’t able to be weaned from mechanical ventilation for ten days. He also was treated with dexamethasone for fifteen days prior to this, she had been healthy. The patient had atrial fibrillation (130 beats /min), tachypnea (28breaths/min) and elevated white blood cell counts (22400/mm^3^) in intensive care unit. General status of the patient got better, weakness and pain in his lower extremities had developed and gradually quadriplegia arose. On musculoskeletal system examination, muscles of bilateral upper extremities, trunk muscles and hip muscles were 1/5, muscles around knees were 2/5 and ankle and toe muscles were 1/5. Light touch, pain, temperature and joint position perception were impaired in distal lower extremities. Deep tendon reflexes were absent. Biochemical analysis was normal except low albumin (3.1 g/dl) (3.5-5.2).

Results of electrophysiologic studies, motor and sensory conduction studies and needle electromyographic studies showed axonal mixed sensorimotor polyneuropathy predominantly in distal lower extremities.

**Fig.1:**
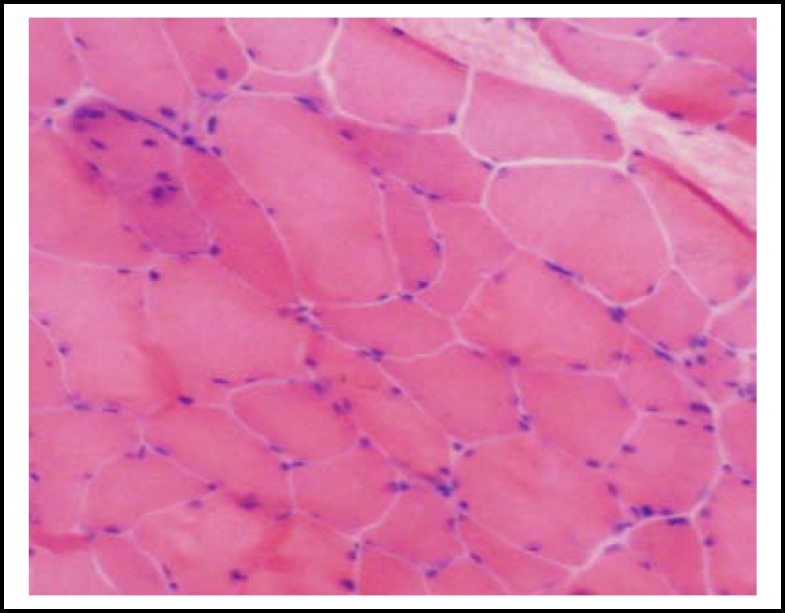
Muscle biopsy of a 40-year-old male CIP patient. HE staining showed atrophic muscle fibers which are mostly angular, scattered, necrotic and regenerative. (x200 magnification). Scale bar=100 μm

***Muscle biopsy*** ***:*** HE staining showed that muscle fiber differ in size and atrophic fibers are mostly angular. Scattered necrotic and regenerative muscle fiber and small blood vessels appeared within muscle and were mildly infiltrated with inflammatory cells (Fig.1).

**Fig.2:**
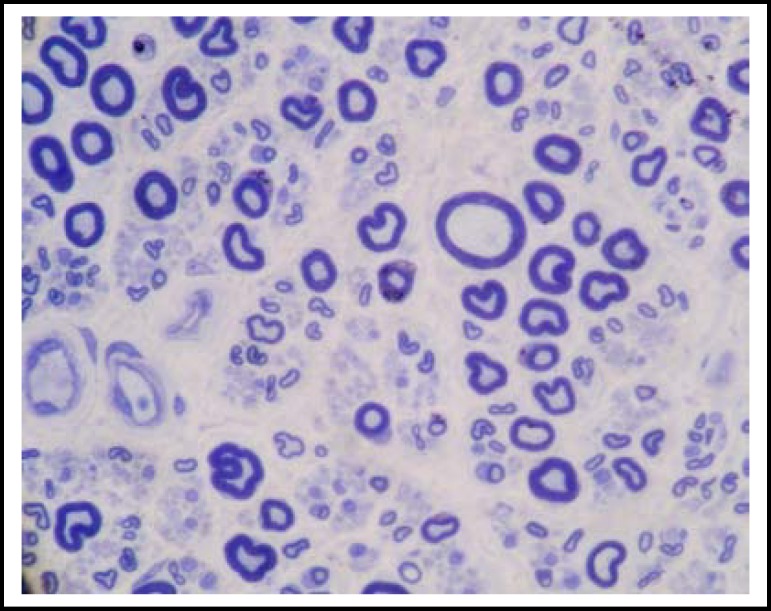
Nerve biopsy of the same patient. Toluidine blue staining showed large-diameter myelinated fibers are significantly reduced. (x200 magnification). Scale bar=100 μm

***Nerve biopsy*** **:** Toluidine blue staining showed large-diameter myelinated fibers are significantly reduced and some nerve fiber swelled (Fig.2). There was no evidence indicating heavy metal poisoning, porphyria or specific vitamin deficiency in our patient. When intensive care unit history, weaning difficulty from mechanical ventilator, clinical manifestations in intensive care unit associated with systemic inflammatory response syndrome (SIRS) (tachycardia, tachypnea and elevated white blood cell counts), symmetrical paresis pronounced in distal lower extremities, absence of deep tendon reflexes, evidence of distal sensory impairment, and presence of electrophysiologic results indicating axonal sensorimotor polyneuropathy were taken into consideration, he was diagnosed as critical illness polyneuropathy according to Bolton’s diagnostic criteria.

## DISCUSSION

Clinical diagnosis of CIP is difficult. The severity of the disease ranges from the presence of electrophysiological abnormalities without any clinical symptoms, to tetraparesis with respiratory insufficiency. Muscular atrophy and distal sensory disturbances are seen in 75% of the patients.^2^^-^^4^ Similar to these findings, our patient was diagnosed as critical illness polyneuropathy. He had intensive care unit history, respiratory failure, mechanical ventilation duration longer than 7 days, clinical manifestations in intensive care unit associated with SIRS, symmetrical paresis pronounced in distal lower extremities, absence of deep tendon reflexes, evidence of distal sensory impairment, and presence of electrophysiologic results indicating axonal sensorimotor polyneuropathy. 

Although seen in all age groups, CIP is rare in childhood. Most of the patients are older than 50 years of age, and it is seen twice more common in males.^4^ Our patient was male, but was in younger age group.

Acute inflammatory demyelinating neuropathy, the most frequent clinical picture of Guillain Barre Syndrome (GBS), and rarely seen acute axonal neuropathy must be kept in mind in the differential diagnosis. In the differential diagnosis of these disorders, it is important that the symptoms of GBS become manifest before their admission to the intensive care unit. GBS is related to *Campylobacter jejuni *infections and anti-ganglioside antibodies, and albuminocytologic dissociation is demonstrated in the cerebrospinal fluid (CSF) analysis.^4^ Electrophysiologic findings of CIP are characterized by fibrillation potentials and positive sharp waves, decreased amplitudes of compound muscle and sensory nerve action potentials.^3^ Needle electromyographic studies show fibrillation potentials and positive sharp waves at rest. The conduction velocity is normal or slightly slow. There is no temporal dispersion or conduction block.^4^ GBS differs from CIP with considerable prolongation of impulse conduction, dispersed compound action potentials or of conduction block. There are much less abnormal spontaneous activity in muscles in GBS which would be seen predominantly demyelinating polyneuropathy. Critical illness myopathy may frequently accompany CIP, and it may cause difficulty in the diagnosis in patients with isolated motor axonal neuropathy; it can be differentiated with serum creatine kinase level, electrophysiological tests and muscle biopsy.^3^ There were no findings of myogenic involvement in the electrophysiologic test and the serum creatine kinase level was normal in our patient, so we did not consider critical illness myopathy.

The disease improves in the mild cases within weeks, whereas improvement takes months in the severe cases.^2^ The mortality rate ranges between 36 and55%.^4^Sepsis, systemic response syndrome and multiple organ failure are the main risk factors. Female gender, severity of illness, duration of organ dysfunction, renal failure, parenteral nutrition, low serum albumin,duration of intensive care unit stay, vasopressor and catecholamine support, central neurologic failure and hyperglycemia have been identified as independent risk factors.^2^ Our patient’s risk factors were long duration of stay in intensive care unit, presence of respiratory failure and clinical manifestations of SIRS, and lower serum albumin level.

With this case report, we draw attention to critical illness polyneuropathy, a disease that clinicians must keep mind in patients with symmetric motor weakness who had been followed up in intensive care unit.
